# Effects of Feedback From Self-Monitoring Devices on Lifestyle Changes in Workers with Diabetes: 3-Month Randomized Controlled Pilot Trial

**DOI:** 10.2196/23261

**Published:** 2022-08-09

**Authors:** Tomohisa Nagata, Sona-Sanae Aoyagi, Minekazu Takahashi, Masako Nagata, Koji Mori

**Affiliations:** 1 Institute of Industrial Ecological Sciences University of Occupational and Environmental Health, Japan Kitakyushu Japan; 2 Department of Occupational Medicine School of Medicine University of Occupational and Environmental Health, Japan Kitakyushu Japan

**Keywords:** mobile health, digital health, diabetes, workers, self-monitoring, BMI, daily activity, randomized controlled trial, smartphone, mobile phone

## Abstract

**Background:**

Although lifestyle interventions are useful in the prevention and management of diabetes, they can be expensive and time-consuming. There is some evidence on the effectiveness of automated mobile technology for health self-monitoring; however, few studies have used such devices in the occupational health field.

**Objective:**

We aimed to examine the effectiveness of a digital self-monitoring device on glucose levels and activity of workers with diabetes in Japan. The primary outcomes were changes in blood glucose levels, and the secondary outcomes were changes in weight and BMI.

**Methods:**

A 2-arm randomized controlled pilot trial was conducted with workers from 23 organizations. The intervention group (n=50) wore an armband activity monitor, a body composition monitor, and a blood pressure monitor for 3 months and received semiautomated weekly email messages tailored to their device data. The control group (n=53) engaged in no self-monitoring. Messages were developed by a physician and a dietician. Postintervention changes in blood glucose levels, weight, and BMI were compared between the intervention and control groups, using blood tests and questionnaires.

**Results:**

At the end of 3 months, the intervention group showed significantly lower blood glucose levels (HbA_1c_: intervention group mean 6.4% (SD 0.3%) vs control group mean 6.6% (SD 0.3%); Cohen *d*=0.7, 95% CI 0.2-1.1; *P*=.009). There were no significant between-group differences in weight and BMI.

**Conclusions:**

Mobile digital self-monitoring was effective in improving blood glucose levels in workers with diabetes. The use of digital health devices is a cost-effective way of implementing health self-monitoring for large numbers of individuals in the workplace. However, due to the large volume of missing values in this study, we need to be careful in interpreting the results, and well-designed intervention studies need to be conducted.

**Trial Registration:**

University Hospital Medical Information Network UMIN000023651; https://upload.umin.ac.jp/cgi-open-bin/icdr/ctr_view_cb.cgi?recptno=R000027244&flwp_key=1008PYbOcXKmk7CAg4Th1FWS

## Introduction

The number of people with diabetes is increasing, and it is a global public health challenge [1]. In Japan, 18.7% of men and 9.3% of women are at high risk of diabetes [2]. Type 2 diabetes mellitus (T2DM) is associated with both genetic factors and modifiable risk factors such as obesity [3] and physical inactivity [4]. Early detection and prevention of T2DM using lifestyle interventions is preferable to treatment. Even a small amount of weight loss (3%) can improve metabolic status [5]. Additionally, higher daily step count (6000-8000 steps/day) can reduce morbidity [6] and mortality [7]. Lifestyle interventions can reduce T2DM incidence or improve diabetes outcomes [8-14]. However, such interventions often rely on coaching by health professionals, whose skills can affect results [11]. Additionally, the cost and time burdens of lifestyle interventions may preclude their availability to all patients with diabetes.

Interest is growing in the potential benefits of automated wearable or mobile technology as a cost-effective way of monitoring health in large populations [15-17]. A recent review indicated that web-based health behavior change interventions are often as effective as face-to-face interventions, particularly if they feature some type of person-to-person human support to provide encouragement and feedback [18].

Wearable technology devices and smartphones have been used to monitor health in workers with various conditions. Some recent reviews indicate that digital mental health interventions improve workers’ well-being and effectiveness [19] and are also effective in preventing and managing diabetes; such tools may be adopted more widely with the growth of medical device innovations [20]. Two core components of mobile health tools are self-monitoring (eg, of diet, blood glucose levels, activity, and weight) and messaging (eg, educational or motivational comments and self-monitoring data feedback or reminders) [20]. Several randomized controlled or pragmatic trials have examined the use of digital health interventions for diabetes [21-28]. However, these studies show inconsistent results for glycemic improvement, and they were conducted in primary health care settings and not in the workplace. There is a small amount of evidence that message-based interventions can improve diabetes outcomes in workers [29], but more studies are needed.

The aim of this study was to examine the effectiveness of a 3-month, digital, semiautomated behavior improvement program on blood glucose levels, BMI, and health-related behavior of workers with diabetes.

## Methods

### Study Design

A 3-month, 2-arm randomized controlled pilot trial was conducted on Japanese workers with diabetes between June 2016 and August 2016. This was a parallel study with a 1:1 allocation ratio.

### Participants

The aim of this study was explained to companies and health insurance unions, and 23 organizations agreed to participate. In Japan, regular medical examinations are conducted once a year, and most companies obtain employee blood glucose measurements (ie, HbA_1c_). Workers with blood glucose measurements within 3 months before the baseline, whose HbA_1c_—as per the National Glycohemoglobin Standardization Program (NGSP)—was 6.5%-7.0 %, were invited to participate. The inclusion criteria were having 20-65 years of age at the time of participation and permission for participation by the attending doctor or occupational physician.

We excluded workers (1) who received regular diabetes treatment and used insulin or hypoglycemic drugs, (2) had stage ≥4 diabetic nephropathy, (3) were on dialysis or hospitalized, and (4) had been diagnosed with malignant tumors in the last 5 years, as well as (5) pregnant or potentially pregnant women. At the beginning of the study, participants were asked if they wished to seek treatment for diabetes and were informed that they could consult with the company occupational physician if they wished.

### Randomization

One of the researchers conducted a block randomization and analyzed the data, but he was not involved in the intervention. Two sets of blocks of four (2 from the intervention and 2 from the control group) random combinations were generated using computer-generated randomization codes. Randomization was performed 7 times (corresponding to 7 recruitment periods).

### Intervention

The transtheoretical model was used to develop the intervention. The individualized, transtheoretical model-based intervention was an effective strategy for weight management in the primary health care setting [30]. The intervention comprised self-monitoring and tailored feedback email messages. We distributed an armband activity monitor (Moveband 2 WMB-02C-K, LB, BR; NTT DoCoMo Inc) [31], a body composition monitor (HBF-254C; Omron Healthcare Co, Ltd) [32,33], and a blood pressure monitor (HEM-7270C; Omron Healthcare Co, Ltd) [32,34] to the intervention group. Participants were asked to wear the armband activity monitor throughout the day and night except when bathing. The activity monitor measured daily steps. We encouraged participants to measure their body weight and blood pressure at least once a day. The body composition monitor measured body weight and BMI. The blood pressure monitor measured systolic or diastolic blood pressure. The data recorded by the 3 devices were merged into 1 data set. Participants could check their activity status via the 3 devices and their own smartphones. We checked participants’ self-monitoring results once a month. If the rate of wearing the activity monitor or measuring the weight was low, we contacted the participants individually and communicated with them to ask them to measure it.

We conducted biochemical examinations (Sunpre Co, Ltd) using a small amount of blood collected from the fingertip [35] for the intervention and control groups at two time points: baseline and after 3 months. To assess the effect of the intervention, the same examinations were conducted with just the intervention group after 1 month and after 2 months from the baseline. The examinations assessed HbA_1c_ (as per NGSP), total cholesterol, high-density lipoprotein cholesterol, low-density lipoprotein cholesterol, triglycerides, total protein, albumin, glutamic oxaloacetate transaminase, glutamate pyruvate transaminase, γ-glutamyltransferase, urea nitrogen, creatinine, and uric acid. For blood collection, participants warmed their hands and wiped the finger with a sterilizing cloth. They made a small puncture at the fingertip and drew blood into a collecting tool. They mailed the specimen to the laboratory. A biochemical automated analyzer (JCA-BM6050; JEOL Ltd) was used to measure biochemical data. Participants were able to access their examination results 1 week later via their smartphones, tablets, or personal computers.

We sent a semiautomated, tailored email message to participants once a week. The content of the messages was developed by a medical doctor and registered dietician based on Prochaska’s transtheoretical theory of health behavior change [36]. The Japanese Ministry of Health, Labour and Welfare’s health check and guidance manual [37] is based on this theory, and we have applied the text of this manual to this study. This theory emphasizes the important role of self-efficacy and tailored interventions to improve health behavior [38,39]. We sent messages according to the number of steps measured by the participants. The messages were designed to motivate participants to walk; for example, if a participant walked more than 10,000 steps a day, they would be praised (eg, “Great! You have focused on your walking. Keep up the good work”). If a participant walked less than 3000 steps, they would get the message “let’s first focus on walking! Try walking 3 days a week, not every day. It is also effective to take a small walk or change the way you walk.” We also added the message about self-monitoring and how to use the equipment. The overall message structure is provided in Multimedia Appendix 1.

Participants in the intervention group were given the above-mentioned measurement devices free of charge.

Participants in the control group were allowed to seek health guidance from their company’s occupational physician or nurse, but we did not record whether such guidance was sought. Participants who had high HbA_1c_ levels after 3 months were advised to seek medical advice.

### Outcome Measures

Demographic information was collected at baseline. Blood tests and questionnaires assessing motivation for changing physical activity were administered to both groups at baseline and 3 months post intervention (Multimedia Appendix 2). The motivation question was related to changes in physical activity. We asked participants the following question: “do you plan to start, or have you already started, lifestyle modifications to increase your physical activity?” There were 3 response options: “I have no plan to change,” “I currently have a plan to change,” and “I already changed my activity.” This question and response options were based on a standard questionnaire used in Specific Health Checkups [40], a detailed manual developed by the Japanese Ministry of Health, Labour and Welfare for medical checkups required by the National Health Insurance Unions [37], and Prochaska’s transtheoretical theory of health behavior change [36]. The primary outcome measure was changes in HbA_1c_ (in mmol/mol or equivalently in % as per NGSP) from baseline to 3 months. The secondary outcomes were changes in weight and BMI.

### Sample Size Calculations

Sample size calculations assumed an overall 2-sided 5% significance level to be distributed equally between the intervention and control groups. A sample size of 41 in each group was estimated to have at least 80% power to detect a difference of 5 mmol/mol of HbA_1c_ (as per NGSP) post intervention, assuming an 8 mmol/mol SD of HbA_1c_ (as per NGSP) and a significance level of .05. Sample size calculations were performed using the Power and Sample Size software program (version 3.1.6; WD Dupont and WD Plummer) [41]. We assumed a dropout rate of 5% and calculated an overall minimum sample size for each group of 46.

### Process Evaluation

For the purpose of evaluating the intervention process, we calculated the average rate per 30 days of available values from the digital devices (armband activity monitor and body composition monitor) in the intervention group. Especially the mean steps per 30 days by the armband activity monitor was described from the first month to the third month for the purpose of confirming whether the behavior of the intervention group continued over the 3-month period. Additionally, the steps were described, stratified by a BMI with the cutoff point of 25 kg/m^2^ based on Japanese obesity criteria [42] and 27.5 kg/m^2^ based on Asian population obesity criteria [43]. A previous study indicated that participants with a higher BMI had lower average daily steps [44].

### Statistical Analysis

All analyses were performed by an intention-to-treat approach. To examine the effect of the intervention, we conducted chi-square tests and 2-tailed *t* tests post intervention. Effect sizes were calculated using Cohen *d* for continuous variables. *P* values <.05 were considered significant. All analyses were conducted using Stata software (version 16.1; StataCorp).

### Ethical Considerations

The occupational physicians of the companies in the study determined that there were no problems with the participation in the study. In addition, the participants were not restricted from receiving regular occupational health services from the occupational health staff of their companies. We planned that if blood pressure measurements were persistently excessively high during the study period, the investigator, a physician, would contact the individual directly and recommend that he or she see a doctor. However, in reality, none of the participants were directly recommended to see a doctor.

The medical research ethics committee of the University of Occupational and Environmental Health, Japan, approved the study protocol (H28-056). Written informed consent was obtained from all participants. This study is registered with the University Hospital Medical Information Network (UMIN000023651). Trial development and reporting was guided by the CONSORT and CONSORT-EHEALTH statements.

## Results

**Figure 1:**
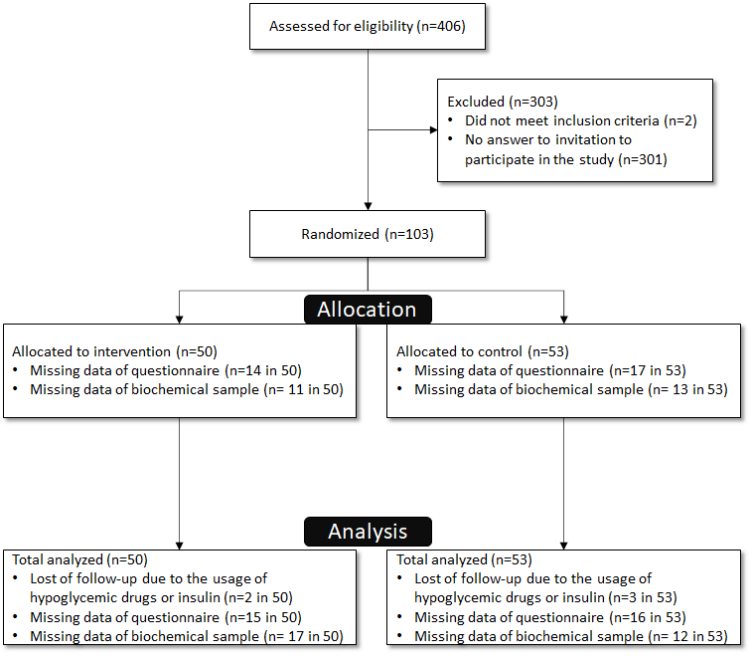
Flow chart of study participants.

Figure 1 shows the flow of study participants. A total of 406 individuals were assessed for eligibility criteria. After exclusions (n=303), 103 participants were allocated to 50 intervention and 53 control groups. Table 1 shows demographics and clinical characteristics at baseline. Among intervention and control groups, 10 (20%) and 8 (15%) were women, respectively.

Post intervention, there was a significant between-group difference in HbA_1c_: the effect size (Cohen *d*) was 0.7 (*P*=.009), and the mean HbA_1c_ for the intervention group (mean 6.4%, SD 0.3%) was 0.2% (2.2 mmol/mol) lower than the mean HBA_1c_ for the control group (mean 6.6%, SD 0.3%; Table 2). There was no significant effect on weight and BMI at postintervention.

In terms of weight change (kg and %) before and after the intervention, the mean weight change for the intervention group was –1.36 (SD 3.6) kg and –1.35% (SD 4.2%), whereas for the control group, it was –0.53 (SD 2.1) kg and –0.72% (SD 2.6%), with no significant difference between the two groups (*P*=.23 vs *P*=.46).

There was a significant between-group difference in motivation for changing physical activity post intervention (*P*<.001).

Additionally, the average rate per 30 days of available values from the digital devices (armband activity monitor and body composition monitor) in the intervention group was very high compared to the control group (84% and 69%, respectively). The mean number of steps per day for the first, second, and third month in 3 months was 7226, 7431, and 7609, respectively (Table 3). Participants with a higher BMI had a lower mean number of steps. Using a BMI cutoff point of 25 kg/m^2^, the mean number of steps remained high for those with a BMI <25. The intervention group experienced no adverse effects.

**Table 1:** Demographics and clinical characteristics at the baseline (N=103).

Characteristics		Participants	
		Control group (n=53)	Intervention group (n=50)
**Sex, n (%)**			
	Women	8 (15)	10 (20)
	Men	45 (85)	40 (80)
Age (years), mean (SD)		52.3 (6.6)	51.5 (7.1)
**Education, n (%)**			
	High school graduation	12 (23)	12 (24
	University degree or higher	26 (49)	24 (48)
	Missing	15 (28)	14 (28)
**Marriage, n (%)**			
	Yes	33 (62)	28 (56)
	No	5 (9)	8 (16)
	Missing	15 (28)	14 (28)
**Job position, n (%)**			
	Manager	17 (32)	13 (26)
	Staff	21 (40)	23 (46)
	Missing	15 (28)	14 (28)
**Occupation, n (%)**			
	Desk work	18 (47)	9 (25)
	Service	5 (13)	7 (19)
	Research and development	6 (16)	5 (14)
	Factory	4 (11)	9 (25)
	Others	5 (13)	6 (17)
	Missing	15 (28)	14 (28)
**Smoking, n (%)**			
	Everyday	10 (19)	13 (26)
	Missing	17 (32)	14 (28)
**Alcohol drinking, n (%)**			
	More than 5 days/week	10 (19)	12 (24)
	Missing	17 (32)	14 (28)
**Body indices, mean (SD)**			
	Weight (kg)	77.7 (13.3)	76.5 (13.6)
	BMI (kg/m^2^)	27.1 (3.7)	27.1 (4.5)
	Missing, n (%)	14 (26)	12 (24)
**Motivation for changing physical activity, n (%)**			
	“I have no plan to change.”	4 (8)	5 (10)
	“I currently have a plan to change.”	19 (36)	20 (40)
	“I already changed my activity.”	13 (25)	11 (22)
	Missing	17 (32)	14 (28)
**Blood tests, mean (SD)**			
	HbA_1c_^a^ (%)	6.5 (0.5)	6.6 (0.5)
	HbA_1c_ (mmol/mol)	47.9 (5.0)	49.1 (5.7)
	FBG^b^ (mg/dL)	129.7 (34.5)	127.7 (35.6)
	Total cholesterol (mg/dL)	203.6 (38.0)	205.0 (36.0)
	HDL^c^ cholesterol (mg/dL)	54.8 (13.6)	51.7 (10.8)
	LDL^d^ cholesterol (mg/dL)	119.0 (29.4)	117.6 (29.0)
	Total protein (g/dL)	7.5 (0.2)	7.4 (0.3)
	Albumin (g/dL)	4.5 (0.1)	4.5 (0.1)
	GOT^e^ (IU/L)	32.2 (9.1)	33.9 (11.4)
	GPT^f^ (IU/L)	30.2 (21.5)	30.5 (12.8)
	γGT^g^ (IU/L)	44.6 (39.3)	53.3 (56.3)
	BUN^h^ (mg/dL)	13.7 (3.5)	13.8 (3.4)
	Creatinine (mg/dL)	0.8 (0.1)	0.7 (0.1)
	Urinary uric acid (mg/dL)	5.4 (1.2)	5.4 (1.0)
Missing blood test data, n (%)		13 (24)	11 (22)

^a^HbA_1c_: hemoglobin A_1c_.

^b^FBG: fasting blood glucose.

^c^HDL: high-density lipoprotein.

^d^LDL: low-density lipoprotein.

^e^GOT: glutamic oxaloacetic transaminase.

^f^GPT: glutamic pyruvic transaminase.

^g^γGT: γ-glutamyl transferase.

^h^BUN: blood urea nitrogen.

**Table 2:** The effect of the intervention compared between the intervention and control groups.

Characteristics		Participants					Effect size, Cohen *d* (95% CI)			*P* value	
		Control group (n=53)	Missing data	Intervention group (n=50)	Missing data						
HbA_1c_^a^ (%), mean (SD)		6.6 (0.3)	12	6.4 (0.3)	17	0.7^b^ (0.2 to 1.1)			.009		
HbA_1c_ (mmol/mol), mean (SD)		48.7 (3.6)	12	46.5 (3.0)	17	0.7^b^ (0.2 to 1.1)			.009		
Weight (kg), mean (SD)		76.5 (13.2)	11	74.8 (13.2)	13	0.1 (–0.4 to 0.5)			.58		
BMI (kg/m^2^), mean (SD)		26.7 (3.7)	11	26.5 (4.4)	13	0.1 (–0.4 to 0.5)			.83		
**Motivation for changing physical activity, n (%)**											<.001
	“I have no plan to change.”	7 (13)		5 (10)		N/A					
	“I currently have a plan to change.”	18 (34)		4 (8)		N/A					
	“I already changed my activity.”	12 (23)		26 (52)		N/A					
	Missing data	16 (30)		15 (30)		N/A					

^a^HbA_1c_: hemoglobin A_1c_.

^b^*P*<.01.

**Table 3:** The trajectory of mean steps per day in the intervention group (N=50).

Characteristics			Participants, n (%)		Mean number of steps per day^a^			
					1-30 days	31-60 days		61-90 days
Total			42 (84)		7226	7431		7609
Missing			8 (16)					
**BMI at baseline**								
	<25	12		9230		9924	9950	
	≥25	23		7047		7294	767	
	<27.5	22		8507		9360	9179	
	≥27.5	13		6602		6391	6330	

^a^SD was not listed due to variations within the same individual and between individuals.

## Discussion

### Principal Findings

This is a randomized controlled pilot trial to evaluate the effects of a digital health intervention on blood glucose, BMI, and health behavior in workers with diabetes. After 3 months, HbA_1c_ levels significantly improved in the intervention group, but this was not the case for weight and BMI. The intervention group also showed greater motivation for changing their daily physical activity.

There was a significant improvement in HbA_1c_ values between the intervention and control groups. HbA_1c_ levels reflect the mean blood glucose level over the past 1-2 months and may reflect the effect 1-2 months after the start of the intervention. Few studies have used glycemic markers as an efficacy index in digital health interventions for diabetes prevention and weight loss. According to recent reviews, the median change in fasting glucose is −0.2 mmol/L [45], and the mean change is −0.1 mmol/L [46]; these changes are smaller than those found in this study. Even a relatively small drop of 0.5% in HbA_1c_ values is clinically important in individuals with values of 6.5-7.0, as reducing HbA_1c_ to <6.5 can help prevent microvascular complications such as retinopathy [47,48]. A previous study [28] of tailored behavior support for physical activity, diet, weight loss, stress coping, and sleep compared blood glucose levels after 6 months of weekly email interventions; HbA_1c_ was −0.26% in the intervention group and −0.18% in the control group [28]. However, another study of a mobile health intervention for patients with diabetes showed no difference in HbA_1c_ values between the intervention and control groups one year later [21]; therefore, evidence is inconsistent, perhaps because of between-study differences in populations and intervention programs. Since the number of steps taken before the intervention is unknown, it is difficult to say for sure, but the number of steps taken by the intervention group increased slightly over the 3-month intervention period (Table3), suggesting that the increase in activity may have led to an improvement in HbA_1c_. The daily self-monitoring of weight using scales may have boosted self-motivation to increase physical activity [49]. The questionnaire responses after 3 months revealed that motivation to increase physical activity had increased significantly more in the intervention group. This motivation may lead to the behavior change of walking. Participants with a BMI of 27.5 kg/m^2^ or higher had a decrease in their mean number of steps over the course of 3 months. How to get people with obesity to be active and how to maintain their behavior is an important public health issue.

There was no between-group difference in the secondary outcome—changes in weight and BMI. The weight reduction among the intervention group was 1.4 kg in 3 months, which is lower than previously reported reductions of 2.3 kg (over 12 months) [46] and 2.4 kg (over 3 months) [50] for diabetes lifestyle interventions. One reason for the lack of weight loss may be inadequate subscription to the diet.

Several randomized controlled trials have examined the use of digital health interventions for diabetes when the HbA_1c_ of participants was more than 7.5 [22] or more than 8.0 [21,24]. The new finding of this study was that intervention in subjects with an HbA_1c_ as low as 6.5-7.0 was effective in reducing HbA_1c_ levels. The interventions in this study were via self-monitoring and semiautomated tailored email messages. However, the latter did not elaborate an individualized message, unlike previous studies that individualized them [20,28]. The actual messages are all shown in Multimedia Appendix 1. Although this is a weakness of the study, it shows that self-monitoring may be effective even without individualized messages. Self-monitoring is an intervention method that requires less professional involvement and is more economical. In the workplace, workers may show the results of their self-monitoring to each other and encourage each other. This will increase the motivation for behavior change and will sustain it.

There were some limitations in this study. First, there were a large volume of missing data in the questionnaires and biochemical tests. It is unclear how the bias of the missing data affects the results. Intervention studies need to be designed and conducted in such a way that missing data will be minimal. Second, only 103 (25%) ultimately agreed to participate, although 406 individuals were recruited by being directly approached about participating in this study. Participants in this study received incentives such as blood pressure monitors, but many workers decided not to participate. This suggests that it is difficult to change people’s behavior. In addition, participants in this study were likely to have a high level of awareness of behavior change, so we need to be careful in interpreting the results of this study. Third, the primary outcome of this study, changes in HbA_1c_ levels, reflects blood glucose levels over the past 1-2 months. The effect of the study should be verified by follow-ups for at least 6 months. However, in this study, we were only able to follow up for 3 months after the start of the intervention due to difficulty in securing a budget for the research project. Since there was an improvement in HbA_1c_ in the intervention group within 3 months, it is possible that a longer follow-up would have resulted in a greater effect. It is necessary to verify the results by long-term follow-ups in the future. Fourth, participants were from relatively large companies, and it is unclear whether the findings can be generalized to workers of small companies. Fifth, as body weight was self-measured, the validity and reliability of BMI values were limited. Sixth, it may be possible that occupational stress may have affected the findings; however, this is unlikely, as there was no difference in occupation and job position between the intervention and control groups.

### Conclusions

The 3-month, digital health intervention for self-monitoring and the semiautomated tailored messages were effective in reducing HbA_1c_ levels in workers with diabetes. This study shows that physical activity can be increased, and blood glucose control can be improved without imposing a professional workload. However, we need to take into account the large number of missing values in this study and be careful in interpreting the results; further well-designed intervention studies need to be conducted. Despite the fact that many people were approached to participate in this study, few actually did so. It may be necessary to develop interventions that would encourage a wider range of workers to participate.

## Appendix

Multimedia Appendix 1 Example of email messages.

Multimedia Appendix 2 Timeline that visually represents the timing of intervention for the intervention and control groups.

## Abbreviations

HbA_1c_: hemoglobin A_1c_
NGSP: National Glycohemoglobin Standardization Program
T2DM: type 2 diabetes mellitus
