# Supplementary material for: Effects of Feedback From Self-Monitoring Devices on Lifestyle Changes in Workers with Diabetes: 3-Month Randomized Controlled Pilot Trial
Source: JMIR Form Res. 2022 Aug 9;6(8):e23261. doi: 10.2196/23261 (PMC9399840; doi:10.2196/23261)
Supplement: Multimedia Appendix 2 [file formative_v6i8e23261_app2.pptx]

## Slide 1
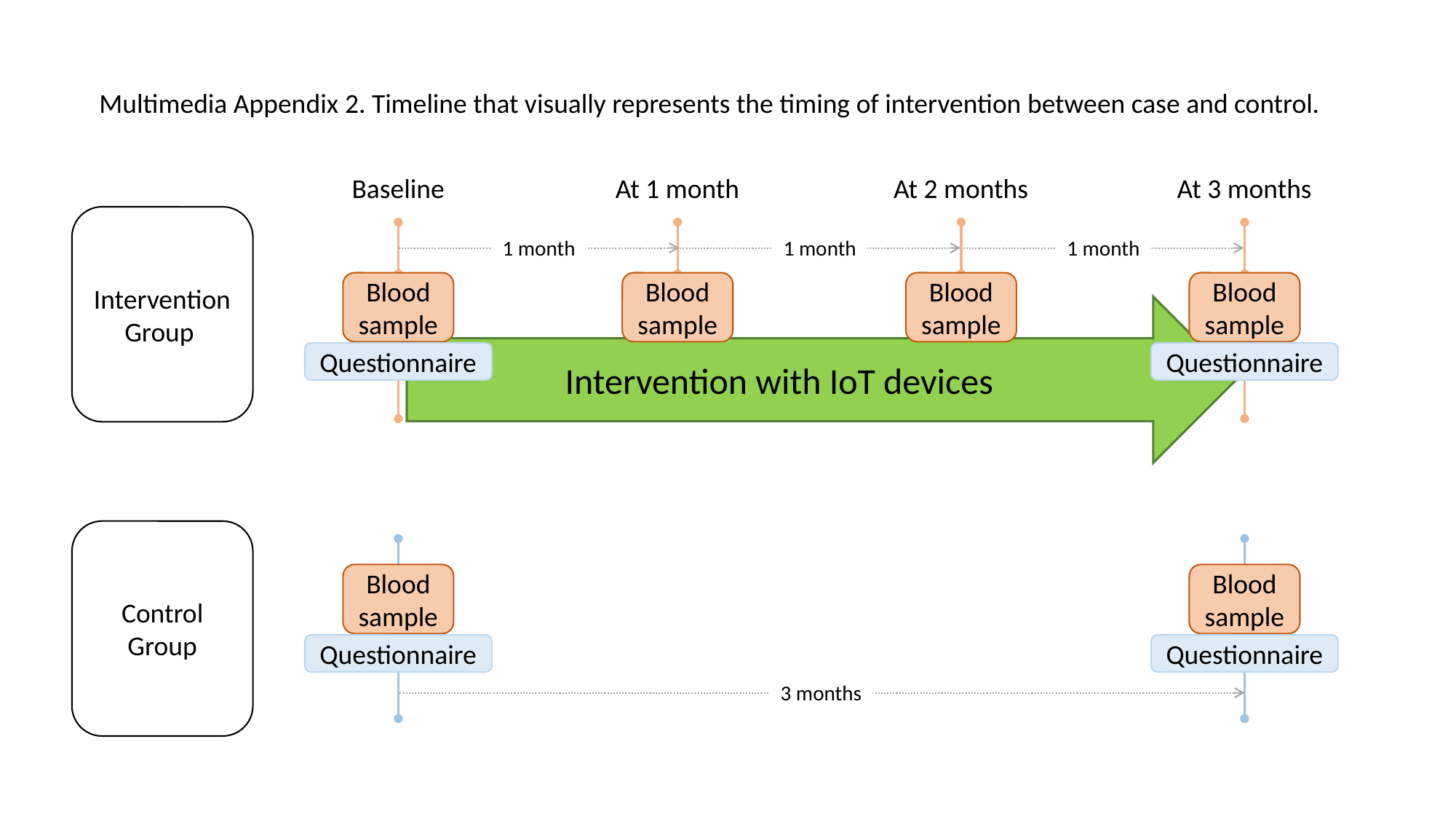

Multimedia Appendix 2. Timeline that visually represents the timing of intervention between case and control.
Baseline
At 1 month
At 2 months
At 3 months
1 month
1 month
1 month
Blood sample
Blood sample
Blood sample
Blood sample
Intervention with IoT devices
Questionnaire
Questionnaire
Intervention
Group
Control
Group
Blood sample
Blood sample
Questionnaire
Questionnaire
3 months
